# Primary Mediastinal Choriocarcinoma in an Elderly Patient with Concurrent Goserelin-Treated Prostate Adenocarcinoma

**DOI:** 10.1155/2019/2734815

**Published:** 2019-05-06

**Authors:** Rasmus Røge, Carsten Simonsen, Astrid Christine Petersen

**Affiliations:** ^1^Department of Pathology, Aalborg University Hospital, DK-9000 Aalborg, Denmark; ^2^Department of Clinical Medicine, Aalborg University, 9000 Aalborg, Denmark; ^3^Department of Cardiothoracic Surgery, Aalborg University Hospital, DK-9000 Aalborg, Denmark

## Abstract

Mediastinal pure choriocarcinomas are exceedingly rare representations of germ cell tumours and are associated with a poor prognosis. To date, fewer than 20 cases have been reported. This current report describes an elderly patient who developed a large rapidly growing mediastinal tumour. Unfortunately, the patient expired before a definitive diagnosis could be reached. An autopsy revealed that the histomorphological features of the tumour showed two distinct tumour cell populations (syncytio- and cytotrophoblasts), and the diagnosis of choriocarcinoma was made. Immunohistochemical analysis showed a characteristic staining pattern in agreement with published studies. Here, we report a case of primary mediastinal choriocarcinoma in an elderly male with concurrent metastasizing prostate adenocarcinoma treated with long-term goserelin deposits, which, as we speculate, could have induced the choriocarcinoma.

## 1. Case Report

Choriocarcinoma is a rare malignant tumour that most commonly develops from the chorionic part of the placenta. In males, it is most commonly seen in the testes as part of a mixed germ cell tumour in young males. Extragonadal occurrence is rare—preferentially in axial structures, such as the mediastinum, retroperitoneum, and brain. The prognosis of mediastinal choriocarcinoma is usually poor, which underlines the necessity of rapid diagnosis [1].

We report here a rare case of primary mediastinal choriocarcinoma in an elderly patient with concurrent goserelin-treated metastasized prostate adenocarcinoma.

A 71-year-old man presented with a 5-week history of severe back pain. Five years earlier, the patient was diagnosed with metastatic prostate adenocarcinoma (Gleason score 4+4) and was treated with gonadotropin releasing hormone (GNRH) agonist implants (goserelin, 10.8 mg). After 9 months of treatment, prostate specific antigen (PSA) values returned to normal levels.

Upon admission, bone scintigraphy showed no bone metastases but a potential compression fracture of the L2 vertebra. CT-scan confirmed a lesion in L2 and revealed a 5-cm spherical tumour located in the anterior mediastinum and multiple lung metastases. The periphery of the mediastinal tumour had high levels of Fludeoxyglucose metabolism as seen on PET-CT. The lung metastases and L2 were also PET-positive. Needle biopsy from the mediastinal tumour showed necrosis but no tumour cells. Decompressive laminectomy was performed, and suspected tumour tissue was sent for histological examination. Unfortunately, the patient expired before any conclusive diagnosis could be reached and therapy instituted.

Autopsy revealed a 5.5 cm spherical tumour in the mediastinum adherent to but not invading the left lung. The lung parenchyma bilaterally contained multiple suspected metastases. The prostate was slightly enlarged but showed no macroscopical signs of adenocarcinoma. Both testes were atrophic and without signs of focal lesions.

**Figure 1:**
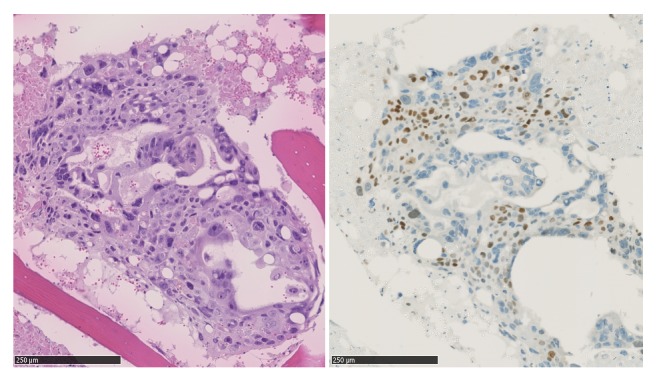
Left: HE stained section of choriocarcinoma metastasis in L2. Right: the same section stained immunohistochemically for p63. Positive reaction (brown) in the nuclei of the cytotrophoblast cells.

The extensive microscopic examination of the mediastinal tumour revealed widespread necrosis and peripheral areas with two distinct tumour cell populations (syncytio- and cytotrophoblasts) (Figure 1). Morphologically, the mediastinal tumour and the metastasis in the lung and lumbar vertebra were a pure choriocarcinoma. The tumour was extensively characterized by immunohistochemistry and showed binary expression of diagnostic markers (Table 1). Microscopic evaluation of the prostate revealed small areas (5% of the total volume) with residual adenocarcinoma.

On the basis of the findings above, we concluded that the tumour was a primary mediastinal choriocarcinoma.

## 2. Discussion

The origin of extragonadal choriocarcinomas has been debated [2]. They have been suggested to develop from foci of pluripotent cells in the axial structures or to be metastases from a testicular tumour. In a case series of eight primary mediastinal choriocarcinomas, no primary lesions were found in the testes suggesting the existence of true primary mediastinal choriocarcinomas [1]. As we unfortunately did not perform microscopic examination of the testes, this case does not shed further light on this issue.

Diagnosis of extragonadal choriocarcinomas can be challenging due to samples comprising predominantly necrosis but only small amounts of tumour cells suitable for diagnosis. Immunohistochemical markers can often promote correct diagnosis.

Our findings are similar to previously published immunohistochemical studies of choriocarcinomas. The tumour was classically positive for hCG. The germ cell markers SALL4 and glypican 3 exhibited a characteristic binary staining pattern (Table 1) [3, 4]. One group found glypican 3 strongly positive in the syncytiotrophoblasts, while the cytotrophoblasts were weakly positive [3]. Although the latter result deviates from our findings, we still observed a binary staining pattern. This difference may in part be explained by a different staining methodology. In agreement with a study of 11 choriocarcinomas, GATA3 was positive in both tumour cell subpopulations [5]. In concurrence with a recent article, syncytiotrophoblast cells were positive for inhibin and CK7, while cytotrophoblast cells were positive for inhibin, p63, and CK7 [6].

At time of diagnosis, the patient was 71 years old. This differs significantly from the typical epidemiology of mediastinal choriocarcinomas, which occurs primarily in younger patients [7]. One might speculate that the long-term treatment with goserelin may have stimulated development of the tumour, especially considering that GNRH receptors are expressed in choriocarcinomas [8]. On the contrary, one* in vitro* study found inhibitory properties of endogenous GNRH in a single choriocarcinoma cell line [9]. However, these results may only be extrapolated to goserelin with caution, since synthetic GNRH agonists are 50 to 100 times more potent than endogenous GNRH [10].

Insights into the potential carcinogenic properties of goserelin and knowledge of expression of GNRH receptors may be utilized in the diagnosis and treatment of choriocarcinomas.

## 3. Conclusion

Extragonadal choriocarcinomas are extremely rare germ cell tumours, and diagnosis requires advanced immunohistochemical analysis. The prognosis is usually poor, which underlines the necessity of rapid diagnosis in order to institute treatment before progression hinders treatment. In this case report, we speculate that long-term treatment with goserelin may have stimulated the development of the choriocarcinoma.

## Figures and Tables

**Table 1:** Immunohistochemical expression profile.

	hCG	Inhibin	OCT3/4	SALL4	GLP3	GATA3	CD71	CK7	p63
Syncytiotrophoblast cells	pos	pos	neg	neg	pos	pos	pos	pos	neg

Cytotrophoblast cells	pos	neg	neg	pos	neg	pos	pos	pos	pos
